# Evaluating the Risk of Prescription Opioid Misuse among Adult Emergency Department Patients

**DOI:** 10.1155/2022/1282737

**Published:** 2022-03-02

**Authors:** Henry W. Young II, Neydric Jean, Joseph A. Tyndall, Linda B. Cottler

**Affiliations:** ^1^Department of Emergency Medicine, University of Florida, Gainesville, FL, USA; ^2^College of Medicine, University of Florida, Gainesville, FL, USA; ^3^School of Medicine, Morehouse College, Atlanta, GA, USA; ^4^Department of Epidemiology, University of Florida, Gainesville, FL, USA

## Abstract

**Background:**

Pain is the most commonly treated symptom in the emergency department, and opioids are often prescribed from the emergency department to treat pain. The American College of Emergency Physicians recommends that providers assess the patient's risk of abusing opioids prior to prescribing opioids. In this study, we use a validated risk assessment tool to assess the risk of opioid abuse among emergency medicine patients and the patients' perceptions of their potential dangers.

**Methods:**

This is an observational study conducted in an academic emergency department (ED). All adults presenting to ED were eligible to participate in the study. Individuals were randomly selected to complete a survey which included the Opioid Risk Tool (ORT) and perceptions of sharing controlled substances.

**Results:**

There were 300 participants in the study. The 18–45-year age group was the most commonly represented group (58%), and nearly two-thirds (63%) of the population was female. The average opioid risk score was 8 or high risk. Individuals that were at high risk of opioid abuse were less likely to dispose of their additional medications appropriately (19% vs. 12%) and were more likely to share their additional controlled medications with family or friends (18% vs. 3%).

**Conclusion:**

The emergency department population is at high risk to abuse opioids. The introduction of safer pain management options should be considered among this high-risk group.

## 1. Introduction

### 1.1. Background

Pain is the most commonly treated symptom among individuals presenting to emergency departments and opioids are commonly prescribed from the emergency department to treat moderate to severe pain [1, 2]. Since 1999, there has been a significant increase in the number of opioids prescribed to treat pain and a concomitant increase in opioid-related deaths [3]. In the U.S., 136 individuals die every day as a result of an opioid overdose [4].

Studies have shown that even a single opioid prescription initiated in the emergency department has been associated with an increased risk of recurrent opioid use in the future [5]. Unfortunately, the emergency department is often targeted by individuals seeking to acquire opioids for nefarious reasons. In a recent survey, 21% of high school seniors who reported using opioids nonmedically reported the emergency department as a source of their opioids [6]. As pain is subjective, determining who is truly in pain as opposed to who is merely stating that they are experiencing pain to obtain opioids for nonmedical use continues to be difficult.

### 1.2. Importance

Assessing an individual's risk of abusing opioids is a commonly accepted practice that is included in the universal precautions of prescribing opioids and recommended by the American College of Emergency Physicians, the Centers for Disease Control and Prevention, and other opioid prescribing guidelines [7–9]. Although there are multiple validated screening tools to assess an individual's risk of abusing opioids, few have been assessed in an emergency department venue [10–12]. At the time of the emergency medicine encounter, there is often limited information known about the patient's past and limited resources such as time. In combination with other external structural factors such as crowding in emergency departments, assessing an individual's risk for opioid abuse has been ignored. Though the American College of Physicians has disseminated a policy for prescribing opioids, no recommendations for the use of specific screening tools to assess opioid risks have been made [8].

### 1.3. Goals of This Investigation

Although it has been postulated that the emergency medicine population represents a population at high risk for opioid misuse, there have been few studies to assess this directly [8]. In this study, we used a validated risk tool to assess the risk of opioid abuse in the adult emergency medicine population as well as perceptions of its potential dangers.

## 2. Methods

### 2.1. Study Design and Setting

This observational study was conducted in the waiting room of a tertiary care level 1 academic emergency department with an annual census of 75,000 visits per year.

### 2.2. Selection of Participants

All individuals in the waiting room who were older than 18 years of age were eligible to participate in the study. Individuals were approached in a randomized manner by assigning each chair in the waiting room a number. A random number generator was used to create a series of numbers each day of the study, which corresponded with the number of the chair to be approached. If no person was in the chair, the next chair on the list was substituted. Individuals who had previously participated in the study or who were less than 18 years of age were excluded.

## 3. Methods and Measurements

Once approached and consented for the study, the Opioid Risk Tool, a validated self-administered opioid risk assessment tool to assess the individual's risk of abusing opioids, was administered via an electronic tablet. A research assistant was present to offer assistance if needed.

The score for the Opioid Risk Tool ranges of 0–26. Scores of 0–3 are considered low risk for opioid abuse. Scores of 4–7 are considered moderate risk, and those of 8 to 26 are considered high risk for opioid abuse. Prescription drug abuse was queried through the question, “Have you ever used prescription drugs that were not prescribed for you or took prescription drugs more than prescribed or for longer than prescribed?” Risk factors for opioid abuse assessed were age 18–45 years of age, male gender, lifetime history of binge drinking, illicit drug use, prescription drug misuse, depression, mental health disorders, and family history of substance abuse, prescription drug abuse, or alcohol abuse. Perceptions of sharing prescribed controlled medications and whether the individual had shared prescribed controlled substances such as opioids, benzodiazepines, or other psychoactive medications were assessed at the end. Data was collected over a 4-week period and analyzed using SAS 9.4.

## 4. Results

### 4.1. Characteristics of Study Subjects

Overall, 368 individuals were approached; 300 agreed to be enrolled in the study yielding a response rate of 82%. As shown in Table 1, most of the sample was 18–45 years old (58%), followed by the over 55 year age group (26%). Females comprised nearly two-thirds (63%) of the total population. The characteristics of the sample included high rates of reported alcohol (58%) and illicit drug abuse (40%).

While males were more likely than females to report alcohol abuse (75% vs. 49%) and illicit drug abuse (52% vs. 33%), females were more likely than males to report depression (43% vs. 27%). There was no difference in the rate of prescription drug misuse or other psychiatric disorders such as attention deficit disorder, obsessive-compulsive disorder, bipolar disorder, or schizophrenia in men vs. women (18% vs. 11%).

### 4.2. Main Results

**Figure 1:**
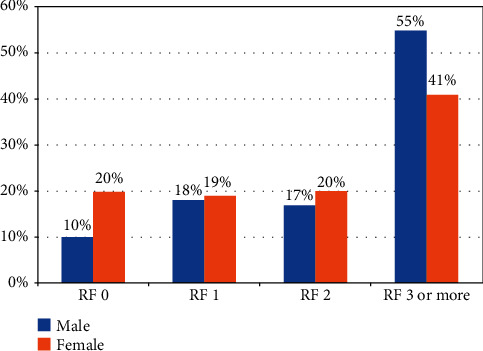
The cumulative number of risk factors (RFs) in the ED population assessed by gender.

As shown in Figure 1, females were more likely than males to have no risk factors (20% vs. 10%), and males were more likely to report three or more risk factors (55% vs. 41%). There were no differences in the proportions of males vs. females with 1 risk factor (18% vs. 19%) or 2 risk factors (17% vs. 20%).

**Figure 2:**
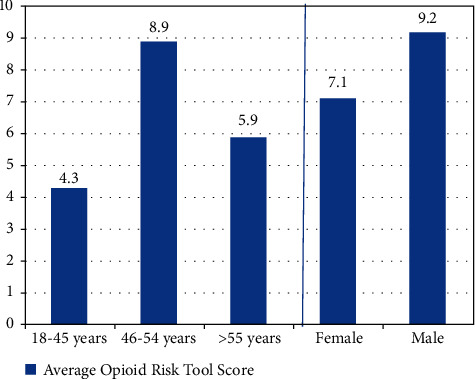
The average Opioid Risk Tool score by age and gender.

As shown in Table 2 and Figure 2, those 18–45 years of age were the most common age range in the high-risk group. When asked about their perception of sharing prescribed controlled substances such as opioids, benzodiazepines, and other psychoactive medications, 15% reported that they perceived nothing wrong with sharing these medications. Overall, almost 1 in 10 (9%) reported that they share their additional prescribed controlled medication with either friends or family. When stratified by risk categories, individuals at high risk for opioid misuse were more likely to share prescribed controlled medications such as opioids, benzodiazepines, or muscle relaxers with friends or family. Individuals with a higher opioid risk score were also more likely to perceive nothing wrong with sharing prescribed controlled psychoactive medications with others.

Overall, the average opioid risk tool score for the emergency medicine population was 8, which is considered high risk for opioid abuse. The age group with the highest ORT score was the 46–54 years group (8.9), followed by the 18–45 years group (8.5), and then >55 year old group (5.9). Males had a higher average ORT score than females (9.2 vs. 7.1) (Figure 2). Overall, nearly half (43%) of the emergency department population was found to be at high risk for opioid abuse, and another 28% were found to be at moderate risk of opioid abuse.

### 4.3. Limitations

Although this included only one clinical site, the population was a randomly selected diverse group who had not been assessed in this manner in the past. The data for the study was self-reported which may introduce bias, as some individuals may be unwilling to share the sensitive information collected in the survey. However, to assess an individual's risk of opioid abuse, sensitive questions are needed, and there were high rates of affirmative responses for sensitive questions in our population. While the ORT has been validated in pain management populations, there is limited evidence validating its use in an emergency medicine population [13]. However, it was the most appropriate means of measurement for our purpose due to its brevity, sensitivity, and ability to be completed autonomously by the patient.

## 5. Discussion

To our knowledge, this is the first study to utilize the Opioid Risk Tool to assess the risk of opioid abuse and willingness to share controlled substances in the emergency medicine population. Overall, the emergency medicine population was found to be at a significant risk of opioid abuse with 43% of respondents being found to have a high risk of abusing opioids. In the Opioid Risk Tool derivation study, 91% of individuals with a high opioid risk score displayed aberrant behavior [10]. The risk this patient population presents is augmented by their also being more willing to share these medications and others with friends or family, and sharing medications with friends and family members was identified as the most common method for obtaining medication for nonmedical use [14].

As shown in previous studies, young adults were the most common age group found to be at risk to abuse opioids [7, 14, 15]. Although the young adult population had the highest rate of individuals at high risk to abuse opioids, the older adult population had the highest average opioid risk score representing a population at the greatest risk to misuse opioids. This is a vulnerable population, as the older adult population is more susceptible to possible adverse effects of opioid medications due to increased medication sensitivity and slower metabolism of these medications [16]. This is augmented by older adults being more likely to have multiple psychoactive medications prescribed to them which may amplify the effects of opioids [17]. Unfortunately, accidental opioid deaths among women in this age group have increased greater than 400% since 1999 [18]. While a great deal of attention has been placed on identifying individuals who seek to use opioids for nonmedical purposes, particularly in the emergency department, much less attention has been devoted to individuals who are at increased risk for adverse reactions to opioids due to their past medical and social history or family history such as this population.

Assessing an individual's risk for opioid misuse is very difficult, especially in the limited time that a provider has in the emergency department and in the absence of a preexisting relationship. Without objective tools such as the Opioid Risk Tool, this vulnerable population and others may be overlooked if not appropriately assessed prior to the prescription of opioids. Screening can allow for the emergency medicine provider to identify individuals at risk if provided opioids and identify individuals who could benefit the most from considering other modalities to treat their pain. Screening individuals in the emergency medicine setting may also allow identification of individuals with substance abuse issues in a population with limited access to care allowing for referral to appropriate treatment. This study also demonstrated that few individuals in the emergency department correctly disposed of medications and were unaware of the dangers of controlled prescription drugs such as opioids. This may contribute to the high rates of sharing of these medications among friends and family that were more pronounced in the high-risk opioid group. This suggests that greater education regarding the potential dangers of opioids is needed among the emergency medicine population.

The ORT is an ideal screening tool for the emergency department as it can be performed in less than 5 minutes, provides ease of interpretation, and provides minimal interruptions in the clinical workflow of the provider as much of the information required for the assessment is already collected in the standard history and physical exam. While Prescription Drug Monitoring Programs (PDMP) have demonstrated usefulness in identifying individuals that have displayed aberrant behavior in the past such as doctor shopping, they fail to identify individuals who are at risk based on their personal psychological history or family history which have been shown to be strong predictors of medication misuse [19]. If the PDMP and the ORT are used together, they can act synergistically to identify individuals at risk for abusing opioids, allow for safer prescribing practices to this high-risk population, and reduce the burden of the opioid epidemic.

In summary, the emergency medicine population was found to be at a significantly higher risk to abuse opioids. The risk of opioid abuse was augmented by those with the highest risk to abuse opioids being more willing to share their controlled substances with others. Future research should assess the impact integration of a validated risk assessment tool into routine emergency medicine care has on prescribing practices and the use of safer pain management options among this high-risk group.

## Figures and Tables

**Table 1:** Gender differences in opioid abuse risk factors among the emergency medicine population.

	Male (*N* = 111) *N* (%)	Female (*N* = 189) *N* (%)	Total (*N* = 300) *N* (%)	*p* value
*Age (years)*				
18–45	71 (64%)	105 (56%)	177 (58%)	0.341
46–54	19 (17%)	25 (13%)	45 (15%)	
>55	21 (19%)	59 (31%)	80 (26%)	

*Family history*				
Alcohol abuse	47 (42%)	71 (38%)	119 (39%)	0.468
Illicit drug abuse	39 (35%)	61 (32%)	100 (33%)	0.688
^*∗*^PRX drug misuse	24 (22%)	37 (20%)	61 (21%)	0.643

*Personal history*				
Alcohol abuse	83 (75%)	92 (49%)	175 (58%)	0.0001
Illicit drug abuse	58 (52%)	62 (33%)	120 (40%)	0.001
^*∗*^PRX drug misuse	18 (16%)	29 (15%)	47 (16%)	0.815
Depression	30 (27%)	81 (43%)	111 (37%)	0.007
Psychiatric disorder	20 (18%)	21 (11%)	41 (14%)	0.087

^*∗*^PRX: prescription.

**Table 2:** The perception of sharing prescribed controlled substances by opioid risk classification.

	Low + moderate risk	Score 0–7	*N* = 171 (%)	High opioid risk	Score 8+	*N* = 129 (%)	Total *N* = 300 (%)	*p* value
*Age (years)*								
18–45	91 (53%)			85 (66%)			176 (59%)	0.03
46–54	24 (14%)			19 (15%)			43 (14%)	
>55	55 (32%)			25 (19%)			80 (27%)	

What do you do with your additional prescribed controlled medications?								
Share w/family and friends	5 (3%)			23 (18%)			28 (9%)	0.001
Save for later	96 (56%)			85 (68%)			181 (60%)	0.11
Dispose of all medications	69 (40%)			29 (22%)			98 (33%)	0.0017
There is nothing wrong with sharing prescribed controlled substances	21 (12%)			24 (19%)			45 (15%)	0.17

## Data Availability

The data used to support the findings of this study are available from the corresponding author upon request.
